# Contemporary and Emerging Therapeutic Strategies in Hypoparathyroidism: From Conventional Treatment to Restoration of Parathyroid Function

**DOI:** 10.3390/jcm15197445

**Published:** 2026-09-24

**Authors:** Dominika Kochan-Olszewska, Aldona Kowalska

**Affiliations:** 1 Department of Endocrinology, Holycross Cancer Center, Artwińskiego 3, 25-734 Kielce, Poland; dominikakochan98@gmail.com; 2 Collegium Medicum, Jan Kochanowski University in Kielce, 25-317 Kielce, Poland

**Keywords:** hypoparathyroidism, PTH, parathyroid hormone, parathyroid diseases, calcium-phosphate metabolism disorders, calcium, phosphate

## Abstract

Hypoparathyroidism (HypoPT) is a relatively rare condition characterised by inadequately low levels of calcium and low levels of parathyroid hormone (PTH), or a complete absence of PTH. In consequence, the body loses its ability to correctly regulate the calcium and phosphate metabolism. Low calcium levels (hypocalcemia) and high phosphorus levels (hyperphosphataemia) are observed in the blood serum of patients. Conventional therapy remains the treatment of choice, involving calcium supplementation (most often calcium carbonate) and active forms of vitamin D, such as alfacalcidol or calcitriol. However, this treatment does not always provide optimal control of the disease or a satisfying quality of life for patients. In recent years, development of therapies using PTH analogues has been observed, as well as intensive research into new pharmacological strategies modulating the PTH/PTH1R signalling pathway. The aim of this paper is to present the current state of knowledge on methods of treatment of hypoparathyroidism and to discuss the prospects for the development of new therapeutic strategies.

## 1. Introduction

Hypoparathyroidism is characterised by hypocalcemia, hyperphosphataemia and an inadequately low level or absence of PTH in the blood [1]. It is a rare disease, with an estimated incidence of 6.4–37/100,000 [2]. Approximately 75% of cases are iatrogenic, most often as a complication of thyroid or parathyroid surgery or other extensive surgical procedures in the neck area, as well as after radiotherapy in this region [1,3]. The remaining 25% of cases are due to genetic or metabolic disorders, autoimmune damage or parathyroid invasion. In some cases, the cause remains unknown [4]. Hypoparathyroidism significantly affects patients’ quality of life, including their physical and mental well-being [5]. Conventional therapy involves the combination of active vitamin D metabolites (calcitriol or alfacalcidol) with calcium supplements. In most patients, conventional therapy does not fully replicate the physiological action of PTH. Despite ongoing treatment, many patients experience unpredictable fluctuations in calcium levels, including episodes of tetany. Furthermore, long-term use of conventional therapy may lead to increased calcium excretion by the kidneys and a further increase in phosphate levels, which in consequence is associated with an increased risk of developing kidney stones, nephrocalcinosis and chronic kidney disease [6,7,8]. The problems associated with treatment described above have a significant impact on patients’ well-being and reduce their quality of life [6].

## 2. Methods

A literature search was performed on PubMed using a range of keywords and combinations related to hypoparathyroidism, its pathophysiology, diagnosis, conventional and PTH-based treatment, complications and emerging therapeutic approaches. The search included combinations of keywords such as “hypoparathyroidism”, “parathyroid hormone”, “PTH”, “calcium”, “phosphate”, “conventional treatment”, “PTH replacement”, “PTH analog”, “hypocalcemia”, “hypercalciuria” and combinations thereof. The relevant literature available up to 2026 was considered, with particular emphasis on recent studies, clinical practice guidelines and expert recommendations. No strict inclusion or exclusion criteria were applied, as this manuscript represents a narrative review. Older publications were included when they provided landmark evidence or addressed established pathophysiological concepts and treatment principles that remain relevant to current clinical practice.

**Figure 1:**
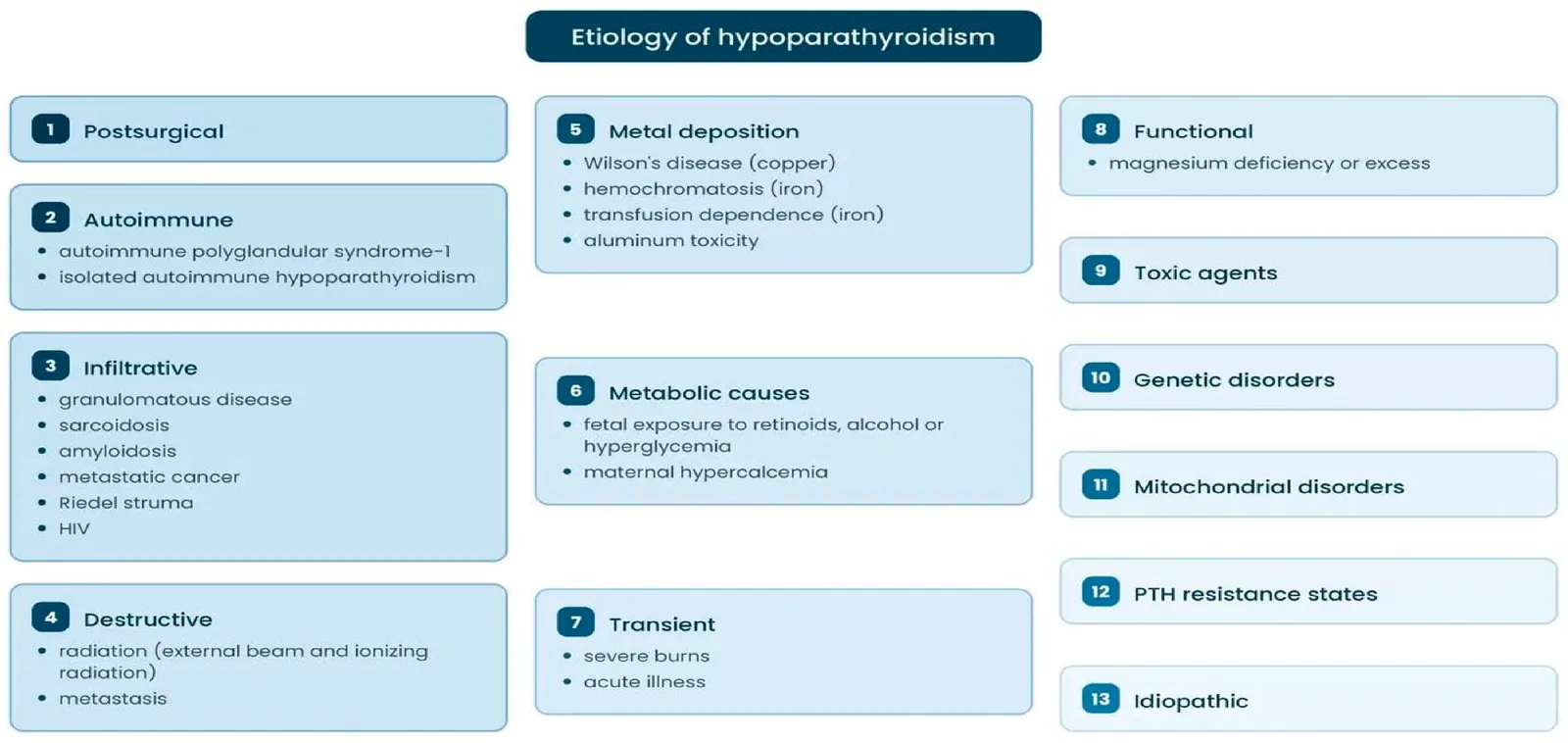
Etiology of hypoparathyroidism [4,9,10,11].

## 3. Treatment of Acute Hypocalcemia

Acute hypocalcemia can be life-threatening and requires urgent intervention. It may present as the first manifestation of the disease in the early postoperative period, as well as in patients already receiving treatment [3]. Despite the chronic nature of hypoparathyroidism, patients may experience episodes of acute hypocalcemia, particularly in the event of sudden changes in calcium and vitamin D requirements, as well as in patients who do not follow therapeutic recommendations correctly. Symptoms of acute hypocalcemia range from mild paraesthesia to painful muscle spasms of the hands and feet and potentially life-threatening complications such as laryngospasm or seizures [12]. The severity of symptoms depends on the absolute serum calcium levels, the rate of decline and the duration of hypocalcemia. In the case of acute, severe symptoms of hypocalcemia, intravenous calcium should be administered. Mild post-surgical hypocalcemia is treated with oral calcium supplements and active vitamin D analogues (alfacalcidol or calcitriol) [3]. Calcium supplementation may also be necessary when the albumin-corrected serum calcium level falls below 7.5 mg/dL (1.9 mmol/L), although there is no universally accepted cut-off point. Intravenous calcium supplementation should be considered regardless of serum calcium level in patients with complications of hypocalcemia, such as symptoms of tetany, laryngospasm, bronchospasm, seizures or prolonged corrected QT interval on ECG (QTc > 0.45 s in men, >0.47 s in women). Intravenous infusion is also indicated in patients who cannot take calcium orally. Therefore, before initiating therapy, albumin-corrected serum calcium and serum magnesium level should be measured and a 12-lead electrocardiogram should be performed to assess the QTc interval. The occurrence of symptoms associated with hypocalcemia, such as cardiac arrhythmias or tetany should also be monitored during treatment [9].

In the treatment of symptomatic hypocalcemia, rapid correction of serum calcium level is crucial. In this clinical context, 10% calcium gluconate is commonly recommended as the first-line treatment [9]. Although calcium chloride provides a higher concentration of elemental calcium in a single ampoule, calcium gluconate is preferred due to the lower risk of soft tissue necrosis in the event of extravasation [1,9]. Treatment begins with a slow intravenous administration of a bolus containing 90 to 180 mg of elemental calcium, which corresponds to 1–2 ampoules (10–20 mL) of 10% calcium gluconate, administered over 10 to 20 min [9,10]. If ampoules of calcium chloride (184 mg/10 mL) are used instead, it should be noted that 1 to 2 ampoules (10–20 mL) provide a significantly higher dose, ranging from 184 to 368 mg (4.6–9.2 mmol) of elemental calcium [1]. An intravenous calcium should always be administered under continuous cardiac monitoring, as rapid calcium infusion is associated with a recognised risk of arrhythmias and sudden cardiac arrest [9,10]. AAs the effect of the initial bolus is transient, a continuous intravenous calcium infusion should be started immediately after its administration. The standard procedure involves diluting 10 ampoules of calcium gluconate (approximately 900 mg of elemental calcium) in 1 L of a 5% dextrose or normal saline. This infusion usually begins at a rate of 50 to 100 mL/h and is continuously adjusted based on frequent measurements of serum calcium concentrations [9,10]. Alternatively, if calcium chloride is used, 5 ampoules (920 mg of elemental calcium) can be diluted in 500 mL of solvent and started at 50 mL/h [1]. An intravenous dose of 15 mg/kg of elemental calcium over 4–6 h can cause an increase in serum calcium level by approximately 0.5–0.75 mmol/L [9].

In previously untreated patients whose acute tetany symptoms have subsided, it is recommended to start oral calcium supplementation at individualized doses, depending on clinical assessment. At the same time, it is worth considering the inclusion of therapy with active metabolites of vitamin D [1].

In the case of patients who have previously undergone calcium therapy, it is necessary to identify the causes of the sudden drop in calcium levels and to adjust the current treatment, to prevent recurrence of hypocalcemia [1].

In the case of hypomagnesemia, it is necessary to correct it, as magnesium deficiency may reduce the effectiveness of hypocalcemia therapy [9].

The primary goal of hypocalcemia treatment is to alleviate its clinical symptoms [1,10,13,14]. Serum calcium levels should be monitored every 6 h and then daily, until stabilisation is achieved, in order to restore calcium levels to just below the normal reference range [9].

## 4. Chronic Treatment of Hypoparathyroidism

The treatment of choice for hypoparathyroidism remains conventional therapy, which includes the supplementation of calcium and active metabolites of vitamin D, as well as the correction of serum magnesium levels [9]. During therapy, systematic monitoring of biochemical parameters is necessary to maintain serum calcium levels within the therapeutic range [2.1–2.6 mmol/L (8.4–10.4 mg/dL)] while minimising the risk of hyperphosphataemia, excessive calciuria and deterioration of renal function [1]. In addition, assessment of bone health is recommended in postmenopausal women and men above 50 years of age [9]. In adult patients, a calcium-rich diet is recommended, along with a reduction in phosphorus intake, which requires limiting foods such as dairy products, legumes, meat, nuts, seeds and processed foods [9].

In certain situations, such as after bariatric surgery, malabsorption disorders or nasogastric tube feeding, conventional therapy may be difficult to implement. Treatment often requires taking large numbers of tablets, which is associated with the risk of side effects and reduced patient comfort. Although conventional therapy is effective in raising serum calcium levels, its main limitation is the inability to maintain stable serum calcium levels. The necessity of strict monitoring and dosage adjustment depending on diet and physical activity makes treatment protocols complex and demanding. Long-term use of conventional therapy may lead to increased levels of calcium in the urine and phosphorus in the serum, which in turn increases the risk of renal complications and ectopic calcifications [9].

### 4.1. Calcium Supplementation

In HypoPT therapy, the daily requirement for elemental calcium is usually between 1.0 and 3.0 g, though some patients may require significantly higher doses to achieve stability [1]. However, supplementation should be individualized and limited to the lowest effective dose needed to maintain serum calcium within the target range [9,15]. Some patients do not require calcium supplementation when they are receiving appropriate treatment with active vitamin D and an adequate dietary intake of calcium [3,15].

The most common recommended calcium supplement is calcium carbonate, which has a high elemental calcium concentration (40%). In certain clinical situations, calcium citrate, which contains 21% of elemental calcium, is used, including patients with achlorhydria, patients on long-term treatment with proton pump inhibitors and people in whom calcium carbonate causes persistent constipation. Another advantage of using calcium citrate is its potential to reduce the excretion of oxalates in the urine [1,9,10,12,16].

The calcium supplementation requirement varies greatly [12]. The intake of calcium supplements with meals improves calcium absorption and at the same time calcium acts as a phosphate-binding agent in the digestive tract, reducing their absorption [3,9,10]. Optimal calcium absorption and stabilisation of calcium and phosphorus levels in blood serum require multiple doses throughout the day [9].

The aim of standard therapeutic management of hypoparathyroidism is to maintain serum calcium levels within the lower range of norm or slightly below the lower limit of reference values, while ensuring that patients do not exhibit clinical symptoms and signs of hypocalcemia [3,10]. In patients who have established a stable treatment regimen with conventional therapy, it is recommended to monitor serum calcium levels approximately twice a year [12]. Regular assessment of renal function and the risk of hypercalciuria forms the basis for monitoring HypoPT. Although specific frequencies vary depending on clinical guidelines, according to the expert consensus, it is generally recommended that 24-h urinary calcium and creatinine excretion should be assessed every 6 to 24 months [9,15]. Annual screening is the generally accepted standard of care for patients in a stable condition [1]. More frequent monitoring should be considered in patients treated with thiazide diuretics or in those requiring higher than generally recommended calcium supplementation, as they are at increased risk of developing hypercalciuria [15]. In addition to laboratory tests, structural assessment of the kidneys is also important for certain groups of patients. In patients with a history of kidney stones or renal calcifications, routine monitoring should be supplemented with imaging studies of the kidneys, such as ultrasound or computed tomography scans, every 3 to 5 years, or immediately if dysuria occurs [1].

### 4.2. Active Metabolites of Vitamin D: Calcitriol and Alfacalcidol

Calcitriol is the most commonly used active vitamin D analogue in the treatment of hypoparathyroidism [17,18]. In some European and Asian countries, alfacalcidol and dihydrotachysterol are used as alternatives. Calcitriol is approximately twice as potent as alfacalcidol [1]. Serum calcium levels typically begin to increase within 12–24 h after calcitriol administration, with stabilisation of the effect after 2–3 days [1]. Although calcitriol has a short plasma elimination half-life of 3–8 h [9], the half-life of its biological effects on calcium metabolism is more than 24 h [15]. This contrasts with alfacalcidol, which requires hepatic 25-hydroxylation, delaying its peak serum concentration to approximately 12 h [9]. Accordingly, in patients previously treated with alfacalcidol, an initial dose reduction of approximately 50% is generally recommended when switching to calcitriol. At the start of treatment or when modifying the dosage of active vitamin D and calcium supplementation, it is recommended to monitor serum calcium levels at 1–2 week intervals, depending on the patient’s clinical condition. It should be noted that increasing the dose of calcitriol or other active metabolites of vitamin D leads not only to an increase in calcaemia, but also to an increase in intestinal absorption of phosphates and an increase in their serum concentration [1].

The use of calcitriol in HypoPT is justified pathophysiologically, as PTH deficiency, often coexisting with hyperphosphataemia, impairs renal conversion of 25-hydroxycolecalciferol D to its active form [19]. The requirement for calcitriol, which is essential for achieving and maintaining optimal serum calcium levels, shows considerable individual variation [1,10,12].

The standard daily requirement for calcitriol ranges from 0.25 to 2.0 µg. To achieve an equivalent therapeutic effect with alfacalcidol, a total daily dose of 0.5 to 4.0 µg is typically required. Regarding administration frequency, calcitriol may be prescribed as either a once or twice daily regimen. Conversely, when utilizing alfacalcidol, it is generally recommended to administer the medication as a single daily dose rather than dividing it throughout the day [15]. Clinical and biochemical assessment is recommended 2–3 days after each dose adjustment [9].

Increasing the doses of active forms of vitamin D may reduce the amount of calcium supplementation necessary to maintain normal calcaemia [12].

It is also recommended to compensate for the deficiency of 25-hydroxyvitamin D and magnesium. Vitamin D supplementation should be focused on achieving a 25(OH)D level of 75–125 nmol/L (30–50 ng/mL). For this purpose, cholecalciferol is administered at doses ranging from 1000 to 100,000 IU per day or 50,000 IU of ergocalciferol once a week or more frequently, including daily dosing, depending on the level of 25(OH)D in the blood. The use of high doses of vitamin D as the only therapeutic intervention (without combined therapy) may lead to significant fluctuations in serum calcium levels and is not recommended [9].

If antiresorptive treatment is necessary in patients with osteoporosis, particular caution is recommended due to the increased risk of hypocalcemia associated with this type of therapy. In order to prevent a decrease in serum calcium levels, it may be necessary to adequately increase the dose of active forms of vitamin D [9].

### 4.3. Thiazides

Thiazide diuretics increase calcium reabsorption in the distal renal tubules and are used in patients with excessive urinary calcium excretion [10,12,20,21]. The use of thiazide diuretics is associated with the risk of electrolyte imbalances, including hypokalaemia, hypomagnesaemia and hyponatraemia [20,21]. In patients with autoimmune polyendocrine syndrome type 1 (APS-1), especially those with concomitant adrenal insufficiency, thiazides may further increase orthostatic hypotension and hyponatraemia. Therefore, this treatment should be administered with particular caution, with regular monitoring of blood pressure and ionogram [1]. Serum magnesium levels should be monitored simultaneously [10,12].

Hydrochlorothiazide is usually administered in doses ranging from 25 to 100 mg per day. The effectiveness of treatment is increased by a low-sodium diet, preferably introduced under the supervision of a dietitian. Studies suggest that the use of thiazide diuretics may be associated with a slight increase in the risk of developing non-melanoma skin cancer, recommendations for sun protection are being made for patients taking medicines from this group [9].

### 4.4. Phosphate-Binding Drugs

In patients with chronic hypoparathyroidism, a phosphate-restricted diet, helps to maintain serum phosphate levels at normal or upper range of normal levels. In cases where inorganic phosphorus levels exceed 6.5 mg/dL, phosphate-binding drugs such as sevelamer, lanthanum carbonate and in exceptional cases, alkalising aluminium preparations are considered [10,12].

In patients with chronic hypoparathyroidism, etiology of hypoparathyroidism is presented in Figure 1, treatment strategies are summarized in Table 1, treatment monitoring is presented in Table 2 and treatment algorithm is shown in Figure 2.

#### Treatment of Hypoparathyroidism During Pregnancy and Lactation

Hypoparathyroidism is quite commonly diagnosed in women of reproductive age. For this reason, both pregnancy and lactation require specialised, multidisciplinary care. During pregnancy and lactation, significant changes occur in calcium and phosphate metabolism, aimed at ensuring proper mineralisation of the fetus’s and newborn’s skeleton, while protecting the mother’s bones [3].

Increased production of PTH-related peptide (PTHrP) by the placenta and breast tissue increases the conversion of 25(OH)D to 1,25(OH)_2_D, which leads to increased absorption of calcium and phosphorus from the mother’s intestines, and may lead to a reduced requirement for calcium and active metabolites of vitamin D in women with HypoPT [3,9]. At the same time, the developing fetal skeleton increases the demand for calcium, and some pregnant women also experience greater calcium loss in their urine. As a result, the tendency to hypocalcemia may increase from early pregnancy, sometimes requiring an increase in the dose of calcitriol to maintain normal serum calcium levels. This risk is particularly significant in women with insufficient calcium intake in their diet [1].

The basis of therapy for pregnant women and women who are breastfeeding with HypoPT remains supplementation with calcium preparations and active vitamin D analogues [1,3,10].

After delivery, the level of 1,25(OH)_2_D quickly normalises. During lactation, PTHrP produced in the mammary glands plays an important role. This peptide increases bone resorption in the mother and increases calcium reabsorption by the kidneys, which usually leads to a reduction in calcium and calcitriol requirements in breastfeeding women with HypoPT. For this reason, during breastfeeding, it is necessary to regularly monitor the albumin-corrected serum calcium level and appropriately reduce the doses of active vitamin D to avoid symptomatic hypercalcaemia [1,9]. After weaning, PTHrP production decreases rapidly and the demand for calcium and active vitamin D increases again. During this period, there is a risk of developing hypocalcemia, which is why strict monitoring of calcium and phosphate metabolism parameters and appropriate modification of treatment are also recommended at this time [10].

Thiazide diuretics are not recommended during pregnancy, as these drugs are classified as category B by the FDA. PTH replacement therapy is also not recommended due to a lack of safety data and is classified as category C [1,10]. Diet is an important factor during pregnancy, but it remains a major challenge for women with hypoparathyroidism. On the one hand, it should contain a balanced amount of protein, carbohydrates, fats, vitamins and microelements and on the other hand, it is important to remember to limit the intake of phosphates to the necessary minimum [1]. In pregnant women and during lactation, serum albumin-corrected calcium levels should be monitored every 2–4 weeks and within 4–7 days after a dosage change, as it is difficult to predict the exact doses of calcium and active vitamin D needed to maintain levels within the recommended range [9]. It is recommended to monitor patients more frequently in the months before and after delivery, as well as when symptoms of hypercalcaemia or hypocalcemia appear. Changes in calcitriol dosage should be monitored by measuring serum calcium levels after 2–3 days [1]. During pregnancy and lactation, it is recommended to maintain serum albumin-corrected calcium levels within the middle or lower range of reference values, with normal serum phosphorus, magnesium and 25(OH)D levels [9]. Due to changes in serum albumin concentration during pregnancy, serum calcium levels should be monitored by measuring albumin-corrected calcium or ionised calcium levels [1]. When planning a pregnancy, treatment should be adjusted to ensure stable serum calcium levels. PTH replacement therapy should be changed to conventional therapy and thiazide diuretics should be discontinued [9]. The aim of treatment is to avoid both hypocalcemia and hypercalcaemia. Calcium deficiency in pregnant women can lead to increased uterine contractility and increase the risk of premature birth or miscarriage. In the fetus, maternal hypocalcemia can cause secondary hyperparathyroidism, which in turn can result in skeletal demineralisation and intrauterine fractures. On the other hand, maternal hypercalcaemia can inhibit fetal parathyroid development and lead to transient hypocalcemia in the newborn [1,3].

## 5. New and Emerging Therapies

### 5.1. PTH Replacement

#### 5.1.1. Parathyroid Hormone in the Treatment of Hypoparathyroidism

For many years, chronic hypoparathyroidism was treated with oral calcium supplementation combined with active vitamin D3 analogues, often in combination with native vitamin D and magnesium. However, more and more data indicate that both the disease itself and long-term use of conventional therapy are associated with the occurrence of significant complications, such as cataracts, intracerebral calcifications, kidney stones and nephrocalcinosis, renal dysfunction, cardiac arrhythmias, ischaemic heart disease, depression and increased mortality [22]. Furthermore, conventional therapy does not fully replicate the physiological action of PTH and may lead to unstable calcium levels, hypercalciuria and hyperphosphatemia, increasing the risk of renal complications and negatively affecting quality of life [6,7,8,12]. These limitations highlight a significant unmet clinical need in the management of chronic hypoparathyroidism and have contributed to the development of PTH replacement therapies. Substitution therapy is a new treatment option that involves correcting the PTH deficiency through the subcutaneous administration of recombinant human PTH [3]. This treatment is particularly recommended in patients requiring high doses of calcium supplements (exceeding 2.5 g of elemental calcium) and active vitamin D (>2 µg/day) to maintain metabolic control, as well as for those in whom even intensive supplementation fails to achieve adequate calcium levels [12]. Alternative treatment should also be considered in patients with renal complications such as hypercalciuria, kidney stones, nephrocalcinosis or renal impairment, as well as in patients with hyperphosphataemia. Additional indications include gastrointestinal disorders leading to impaired absorption and a significant reduction in quality of life associated with the disease [3,6]. PTH replacement therapy should also be considered in patients with poor compliance or intolerance to high doses of calcium and active vitamin D [10].

In 2018, rhPTH 1-84, previously indicated as a treatment for postmenopausal women with osteoporosis [23], was approved by the European Medicines Agency as NATPAR/NATPARA for adults with chronic hypoparathyroidism that cannot be properly controlled with conventional therapy only [3]. In 2019, production of rhPTH (1–84) was suspended by the FDA (Food and Drug Administration) due to the potential presence of rubber particles from the cartridge membrane [9,10].

A new alternative treatment, TransCon PTH (palopegteryparatide, approved under the trade name YORVIPATH), has recently been developed as a long-acting PTH (1–34) prodrug conjugated to polyethylene glycol, which extends the half-life of PTH to approximately 60 h, providing stable PTH levels for 24 h [6,9,24,25]. The EMA (European Medicines Agency) and the FDA have approved palopegteriparatide as a PTH replacement therapy for patients with hypoparathyroidism [9,22].

Synthetic PTH (1–34) effectively increases serum calcium levels, reduces urinary calcium excretion and increases phosphate excretion [10].

The recommended starting dose of palopegteriparatide in adults is 18 µg administered subcutaneously once a day with a gradual dose adjustment of 3 µg every 7 days. The dose should be adjusted individually based on serum calcium levels. The optimal dose after adjustment is the minimum dose required to prevent hypocalcemia. This is the dose that maintains serum calcium levels within the normal range without the need for supplementation with active forms of vitamin D or calcium beyond the recommended dietary supplementation for the general population [9].

Siggelkow and other authors suggest that patients with previous doses below 50 µg rhPTH (1–84) should start treatment with a dose of 15 µg TransCon PTH, patients with 50 or 75 µg rhPTH (1–84) should remain on 18 µg TransCon PTH at the start of treatment, and those with previous doses above 75 µg rhPTH (1–84) should start treatment with 21 µg TransCon PTH before changing treatment. Calcium control remains crucial for 7 days after dose change for all patients [22].

The Phase 3 PaTHway study showed that TransCon PTH is effective, safe and well tolerated by adults with hypoparathyroidism. The results of the study showed a sustained improvement in patients’ quality of life, physical functioning and well-being [6,8]. This study showed that palopegteriparatide enabled independence from conventional therapy while maintaining normal serum calcium and phosphate levels, normalising 24-h urinary calcium excretion and improving bone dynamics and renal function. Most side effects occurring during treatment were rated as mild to moderate. None of the participants stopped using palopegteriparatide due to treatment-related side effects up to week 104 of the PaTHway study [8]. A review of studies comparing PTH therapy with conventional therapy showed a significant reduction in tablet intake after starting treatment with palopegteriparatide, with the greatest reduction occurring within the first 3 months of treatment [10,26].

In order to ensure a safe and smooth transition from PTH analogue therapy to conventional treatment, it is necessary to gradually modify both treatment regimens under strict biochemical control. The dose of PTH (1–34) should be reduced slowly over several days, as sudden discontinuation may result in hypocalcemia. At the same time, the dose of conventional treatment should be gradually increased, taking into account the fact that the demand for calcium and active metabolites of vitamin D in the period after the end of PTH therapy is usually higher than before its initiation, which is associated with persistent intense bone remodelling. During the transition phase, daily measurement of serum calcium levels is recommended, preferably corrected for albumin concentration, especially in patients with previously unstable calcium metabolism, in order to avoid both hypocalcemia and hypercalcaemia and to allow for ongoing adjustment of conventional treatment dosages [9].

A limitation of this therapy may be the need for daily subcutaneous administration, which, for some patients, may be associated with a reluctance to receive injections and inconveniences related to the use and storage of the drug delivery device [27]. Moreover, this treatment is associated with high costs, which limits its widespread accessibility for patients. However, further observational studies are necessary before its widespread use can be recommended, given the limited clinical data available regarding its long-term safety and efficacy [28]. Yet, treatment with palopegteriparatide may reduce healthcare and indirect costs, although this requires confirmation in clinical practice [29]. Despite FDA approval of palopegteriparatide, important gaps remain, including limited long-term safety data, especially on bone health, and a lack of cost-effectiveness and drug-interaction studies. Further research is needed to assess its long-term safety, value, and accessibility [30].

The key differences between conventional therapy and PTH replacement therapy are summarized in Table 3.

#### 5.1.2. MBX 2109

The PTH prodrug MBX 2109 (canvuparatide) was assessed in phase 1 clinical trials. This molecule has a long half-life between 184–213 h, which would justify administering it once a week [31,32]. In the phase 1 study, canvuparatide was generally well tolerated. The most common TEAEs were injection-site reactions, while mild, transient hypercalcemia was a predictable on-target effect. The doses of MBX 2109 have been determined for further studies [31]. Canvuparatide is a promising PTH therapy with the potential for controlled, prolonged release, providing a pharmacokinetic profile similar to continuous PTH delivery without the need for a pump [32].

### 5.2. PTH1R Receptor Agonists

Eneboparatide is a 36-amino acid hybrid PTH/PTHrP peptide intended for parenteral administration, characterised by a long-lasting biological effect. Despite its short pharmacokinetic action time, it has a prolonged intracellular effect. It has been shown that the PTHR1 agonist increases serum calcium levels for more than 24 h after injection and reduces serum phosphorus levels in the HypoPT model in rats after thyroparathyroidectomy. A similar long-term effect on calcium metabolism has been confirmed in studies in monkeys [33].

Eneboparatide, an experimental PTH1 receptor agonist peptide used in the treatment of chronic hypoparathyroidism, underwent a phase II open-label study to evaluate the efficacy, safety and tolerance of the treatment. The drug was administered subcutaneously once a day. After 3 months, more than 88% of patients achieved independence from conventional therapy, while maintaining serum calcium levels within the target range, with simultaneous normalisation of urinary calcium excretion and restoration of balanced bone turnover [34].

The main limitation of eneboparatide is that it has not yet been approved for clinical use. Furthermore, there are still insufficient data to assess its long-term safety and efficacy, while limited experience with its use in routine clinical practice makes it difficult to fully determine its real-world therapeutic profile [35].

The phase II CALYPSO trial showed that eneboparatide was well tolerated, enabled most patients to discontinue conventional therapy, maintained serum calcium within the target range, reduced urinary calcium excretion and supported a return toward physiological bone turnover [34]. The FDA has granted fast-track designation to eneboparatide for the treatment of hypoparathyroidism [9]. The phase III CALYPSO trial evaluated the efficacy and safety of eneboparatide in adults with chronic HypoPT and demonstrated positive results and clinical benefits for patients with HypoPT [36].

An oral small molecule, agonist of the PTH1 receptor is also currently being evaluated as a potential treatment for HypoPT [9].

### 5.3. Calcilytic Therapy

CaSR (Calcium-Sensing Receptor) is a receptor sensitive to calcium levels in the blood. It is present in large concentrations in the parathyroid glands and renal tubules, where it plays an important role in regulating calcium homeostasis. An increase in serum calcium concentration activates the receptor in parathyroid cells and reduces PTH secretion and increases calcium excretion in urine, which helps maintain serum calcium concentration within the normal range [37].

Autosomal dominant hypocalcemia (ADH) type 1 is a rare form of hypoparathyroidism caused by heterozygous activating mutations in the CaSR gene [38]. As a result, PTH synthesis and secretion are inhibited. Patients experience reduced PTH levels, as well as hypocalcemia, hyperphosphataemia and hypomagnesaemia [39,40,41]. Increased calcium excretion in urine results from both reduced PTH level and CaSR activation in the renal tubules [39]. Hypercalciuria is intensified by conventional treatment and is more marked than in other forms of hypoparathyroidism, leading to kidney stones, nephrocalcinosis, chronic kidney disease or cerebral calcification [41,42]. Currently, research is being carried out on the use of CaSR antagonists (calcilytics) as a potential therapeutic strategy in ADH [39,40]. Calcilytics are negative allosteric modulators of CaSR that increase PTH levels in a dose-dependent manner and effectively reduce urinary calcium levels. The study evaluated the pharmacokinetics and pharmacodynamics of the calcilytic compound NPSP795 and confirmed its efficacy and safety [38].

In an open-label phase 2b study (NCT04581629), the oral calcilytic encaleret was administered to 12 patients with ADH1. The drug was administered twice a day, with calcitriol and calcium preparations discontinued. Treatment with encaleret resulted in normalisation of serum calcium, reduction in hypercalciuria and increases in PTH, magnesium, and 1,25-dihydroxyvitamin D levels, while phosphorus levels and tubular phosphate reabsorption decreased. The drug was well tolerated and no serious side effects were reported. This study demonstrated that calcilytics may be a potential treatment for ADH1 [42].

Encaleret is currently being evaluated in phase III trials for the treatment of ADH1 (NCT05680818). Due to its significant effect on renal calcium regulation, this drug is also being studied in a phase II trial in patients with post-operative hypoPT (NCT05735015). The aim of this study is to determine whether encaleret can reduce the urinary calcium excretion fraction and increase serum calcium levels in patients who do not have a mutation in the CaSR gene [9]. Further evaluation of efficacy and safety requires randomized controlled trials involving larger patient populations and longer follow-up periods [43].

### 5.4. Restoring Endogenous PTH Secretion

#### 5.4.1. Parathyroid Transplantation

Parathyroid autotransplantation is considered as the most effective alternative method of treatment to replacement therapy, significantly reducing the risk of permanent hypoparathyroidism. Allotransplantation is an alternative for patients in whom autotransplantation is not possible. The limitations of this method include the possibility of transplant rejection and the need for systemic immunosuppression [44].

In clinical practice, immediately after excision, parathyroid tissue is cooled in a 0.9% NaCl solution at 4 °C for approximately 30 min, which facilitates its preparation. It is then divided into fragments approximately 1 mm thick and implanted into the selected skeletal muscle. Alternatively, it is possible to administer a suspension of fragmented parathyroid tissue in a buffered saline solution, most often into the sternocleidomastoid muscle [44].

Another promising strategy in the treatment of endocrine diseases is microencapsulation, which involves enclosing cells in semipermeable biomaterial shells. Despite its potential, this technique has not yet been introduced into routine clinical practice for the treatment of hypoparathyroidism. The limitations result from the lack of clear data on the optimal number of microcapsules, the number of cells necessary to achieve a therapeutic effect, and the selection of the most favourable anatomical location for their implantation. Further research is needed to determine the efficacy and safety of this method of treatment and to enable its clinical application [44].

#### 5.4.2. Stem Cell Therapy

Although hormone replacement therapy effectively controls the symptoms of hypoparathyroidism, it does not restore endogenous parathyroid function and typically requires lifelong treatment. Therefore, approaches aimed at restoring functional parathyroid tissue, rather than merely replacing missing hormones, represent a promising area of research. In this context, regenerative medicine and stem cell-based therapies may offer a potential alternative to lifelong pharmacological treatment, providing a way to restore endogenous parathyroid function. The results of previous studies indicate that the use of mesenchymal stem cells may be a potential therapeutic strategy in hypoparathyroidism. In the future, it may be possible to obtain functional parathyroid cells by reprogramming fibroblasts or peripheral blood cells from the patient and differentiating them into parathyroid cells in vitro, followed by transplantation of the received cells to restore normal parathyroid function [44].

The study resulted in the creation of parathyroid organoids from human pluripotent stem cells. Molecular analyses confirmed the expression of key markers characteristic for parathyroid tissue [45]. Cells capable of differentiating into parathyroid cells include embryonic stem cells, thymus epithelial cells and tonsil mesenchymal cells. According to some researchers, adipose-derived stem cells also belong to this group [46].

The main advantage of this technology, compared with conventional treatment, is the potential ability of bioengineered tissues to respond dynamically to changes in extracellular calcium levels and to continuously and controllably release PTH. This could help eliminate the chronic and serious complications associated with traditional treatment [44,47].

Despite the promising proof of concept, this method has significant technological and biological limitations. The main problems include the low efficiency of the protocol for producing cells that express PTH, which is only 16.96%, as well as the functional instability and immaturity of the cells obtained. In addition, there is a major gap in knowledge about long-term safety because there is no real-world clinical evidence. There is also limited information about the survival, distribution in the body and potential of the remaining undifferentiated cells to form teratomas [47].

These findings indicate the potential use of cell therapy in the treatment of HypoPT, but further research is needed [45,46,48,49]. Evidence base remains predominantly preclinical, while clinical experience with parathyroid transplantation is limited to small cohorts and case reports [48].

In terms of economics and regulatory approval, it is currently impossible to determine whether this method would be profitable, reimbursed, or widely available because the research is still at a very early, preclinical stage. The method would need to go through a long regulatory process, including strict toxicity tests, tests to confirm genetic stability and several stages of clinical trials. There are also important ethical issues related to obtaining and modifying human embryonic stem cells.

There are also an increasing number of reports indicating that combining cell therapy with biohybrid implants may be a particularly promising direction, which could enhance the effectiveness of HypoPT treatment in the future [44].

#### 5.4.3. Other Regenerative Strategies

Future research directions also include the development of new microcapsule dosage forms, gene therapy and regenerative medicine (e.g., bioengineered thyroid) [46].

The modes of action of new and emerging therapies for chronic hypoparathyroidism are shown in Figure 3 and their comparison is presented in Table 4.

## 6. Discussion

Conventional therapy, which includes calcium supplementation and the use of active metabolites of vitamin D, remains the primary treatment for chronic hypoparathyroidism. Its main goal is to maintain calcium levels at the lower part of the reference range or slightly below its lower limit, while minimizing hypercalciuria and hyperphosphatemia [6]. Despite its widespread use, this approach does not replicate the physiological action of PTH and may be associated with fluctuations in calcium levels, a high number of required tablets and persistent disturbances in calcium-phosphate metabolism [50]. Unlike conventional treatment, PTH replacement therapy aims to correct the underlying hormonal deficiency that causes the disease. For this reason, PTH replacement therapy can be considered an approach that more closely mimics the physiological mechanism of calcium-phosphate homeostasis [9]. Another significant advantage of PTH replacement therapy may be its more favorable effect on certain biochemical parameters, particularly phosphate levels and urinary calcium excretion. Furthermore, recent data on the long-acting PTH analog palopegteriparatide suggest the potential for more stable control of calcium and phosphate metabolism, a reduction in the need for conventional treatment and an improvement in quality of life [9,50]. At the same time, PTH replacement therapy also has certain limitations that must be taken into account when evaluating its role in the treatment of chronic hypoparathyroidism. The availability of this therapy and clinical experience with its long-term use remain more limited than with conventional treatment, and data on its long-term safety, impact on organ complications and clinically relevant endpoints are still insufficient. Furthermore, the benefits observed in studies of individual PTH analogs should not be automatically generalized to the entire class of PTH replacement therapy [10,51]. At the same time, the benefits of PTH replacement therapy should not be interpreted as justification for its routine use in all patients with chronic hypoparathyroidism. Current guidelines continue to recommend conventional treatment as the first-line therapy and suggest PTH replacement therapy primarily for patients in whom standard treatment is insufficient or poorly tolerated [6,9]. Importantly, the recommendation for conventional therapy as a first-line treatment is based on evidence considered to be of low quality [6,10]. Thus, the key difference between the two strategies is not merely their effectiveness in normalizing calcium levels, but above all the way in which they achieve this goal. Conventional treatment corrects the consequences of PTH deficiency by increasing the supply of calcium and active vitamin D, whereas PTH replacement therapy directly compensates for the missing hormonal signal. From the perspective of long-term treatment, this may support the broader use of PTH replacement therapy in appropriately selected patients. However, further studies are needed to evaluate its long-term efficacy, safety and impact on clinically relevant endpoints [8]. Although conventional therapy remains the first-line treatment for most patients with hypoparathyroidism, it has certain physiological limitations [52,53]. Although it effectively corrects hypocalcemia by increasing intestinal calcium absorption, it does not restore PTH dependent renal calcium reabsorption or normal phosphate excretion [53]. This structural pathophysiological defect means that some patients, despite achieving biochemical stability in serum, are at risk for chronic hypercalciuria, nephrocalcinosis, kidney stones and the progression of chronic kidney disease [52,53]. The current medical paradigm therefore aims to replace the missing hormone through PTH replacement therapy, with the goal of lowering urinary calcium levels, reducing the burden of supplementation and improving patients’ quality of life [53].

Despite promising avenues for innovation, a number of unresolved clinical questions and research priorities continue to emerge in the scientific literature that require urgent attention [35,52,53]. A key gap in current medical knowledge is the lack of long-term real-world evidence that would clearly confirm whether new PTH-based strategies effectively reduce hard endpoints, such as the risk of bone fractures, the progression of kidney failure or cognitive decline [35,53]. The impact of substitution therapy on bone metabolism and its long-term safety is still being evaluated in longitudinal studies [52,53]. Another significant challenge is the optimal management of specific patient groups. The transition between conventional therapy and PTH replacement therapy, as well as issues related to pregnancy, lactation and pediatric care, remain areas where high-quality scientific evidence is lacking. Current guidelines must rely almost exclusively on a limited expert consensus [9]. The clinical situation is exacerbated by regulatory and financial differences across jurisdictions. The off-label use of teriparatide is suboptimal and lacks long-term evidence, and hypoparathyroidism in some countries, such as Italy, remains the only endocrine disorder without access to reimbursed hormone replacement therapy. This situation results in a persistent failure to meet patients’ needs, drastically reduces their quality of life, and limits physicians’ ability to prevent irreversible renal and skeletal complications [54]. In view of these challenges, future research priorities should focus on several key pillars, among which the development of medical technologies could prove to be a revolutionary step. Due to unpredictable fluctuations in calcium levels caused by physical exertion, infections, or other factors, patients urgently need tools to monitor changes in blood calcium levels in real time [55]. The next goal should be to develop home-based, point-of-care calcium testing and continuous calcium monitoring sensors. Combined with a PTH delivery pump, these technologies could create a groundbreaking “artificial parathyroid” closed-loop system that would automatically and dynamically adjust the drug dose to maintain stable calcium levels [52,55]. At the same time, translational research must accelerate the development of innovative molecules that go beyond daily injections, such as long-acting prodrugs, oral PTH1R agonists and cell therapy-based strategies aimed at fully restoring physiological mineral metabolism [52]. The future of personalized medicine in this area also depends on advances in the genetic characterization of non-surgical hypoparathyroidism, which will facilitate early diagnosis, accurate risk stratification and the tailoring of treatment to each patient’s comorbidities [35]. Last but not least, a key priority is to optimize care through the education of physicians and patients and the implementation of shared decision-making, which is the foundation for improving adherence to recommendations and tangibly improving patients’ quality of life [52].

## 7. Summary

Hypoparathyroidism is a relatively rare condition that significantly reduces quality of life. Accurate identification of the etiology is essential for implementing optimal treatment. Early diagnosis of HypoPT and the initiation of effective treatment using calcium and active metabolites of vitamin D help to alleviate symptoms. Unfortunately, conventional therapy is often associated with large fluctuations in serum calcium levels and consequently a poor quality of life. Moreover, it does not fully reproduce the physiological actions of endogenous PTH and may be associated with a substantial treatment burden and persistent complications in some patients. In these patients, therapy with PTH analogues may bring promising results. PTH replacement is currently considered an important therapeutic option for selected patients who remain inadequately controlled or experience a high treatment burden despite optimized conventional therapy, although its long-term clinical benefits and optimal use in different patient populations require further clarification.

Therefore, further studies comparing novel treatment strategies with standard therapy are necessary, particularly with regard to long-term clinical outcomes and safety. The dynamic development of pharmacotherapy for hypoparathyroidism also includes new molecules that are currently in advanced stages of clinical development. Novel therapeutic approaches, particularly therapies aimed at PTH replacement or CaSR modulation, offer a realistic opportunity to further improve metabolic control and quality of life in patients with hypoparathyroidism. Their potential clinical significance includes more physiological regulation of calcium–phosphate homeostasis, reduced dependence on calcium supplementation and active vitamin D metabolites and a decrease in daily fluctuations in serum calcium levels. Further research is needed to elucidate the mechanisms underlying complications associated with chronic hypocalcemia, to assess the long-term effects of PTH replacement therapy on target organs and to develop technologies enabling continuous monitoring of serum calcium levels, which may significantly improve treatment individualization in the future. Future studies should also determine which patient groups derive the greatest benefit from specific novel therapeutic strategies and whether their potential metabolic benefits translate into sustained improvements in clinical outcomes and quality of life.

## Figures and Tables

**Figure 2:**
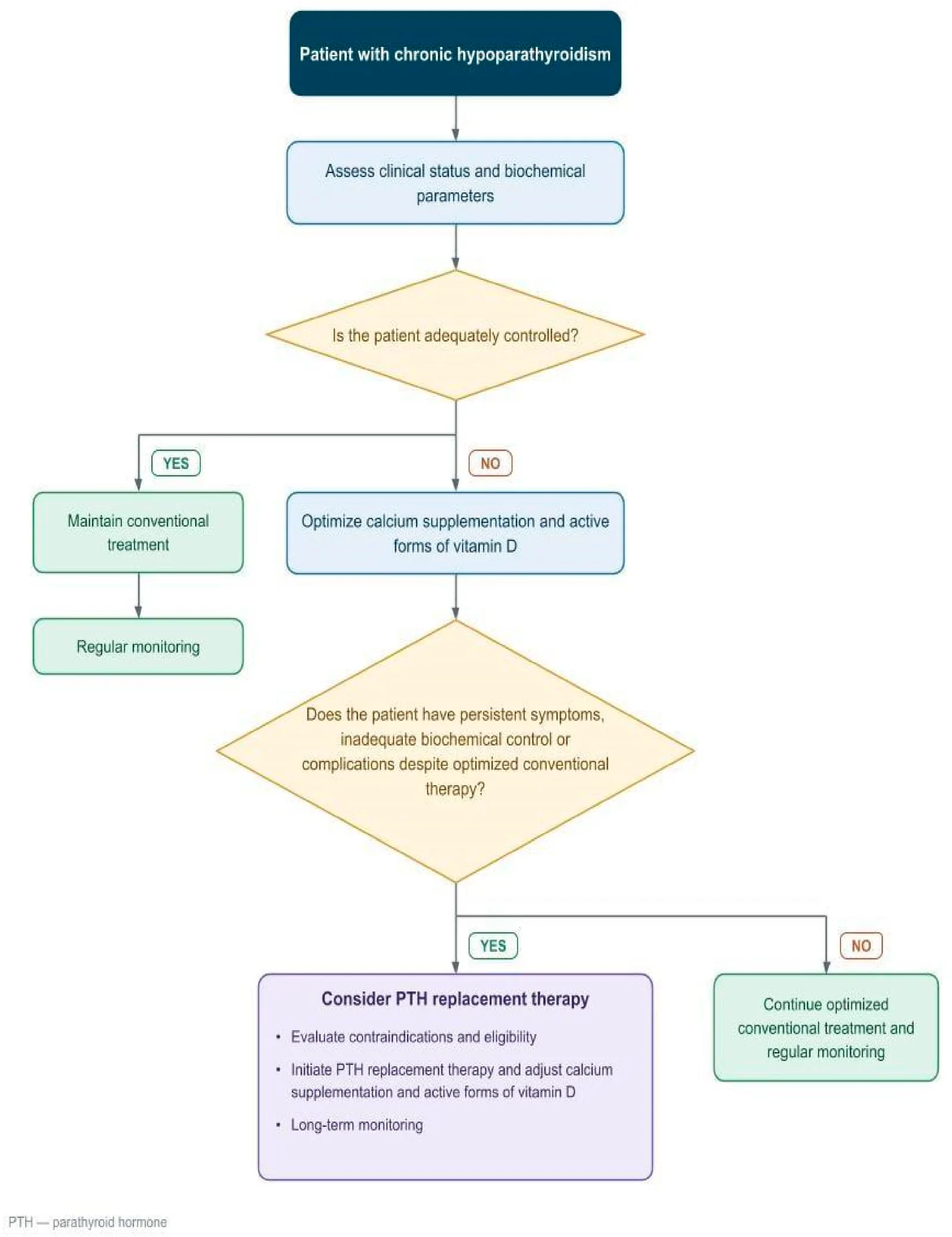
Treatment algorithm for patients with chronic hypoparathyroidism.

**Figure 3:**
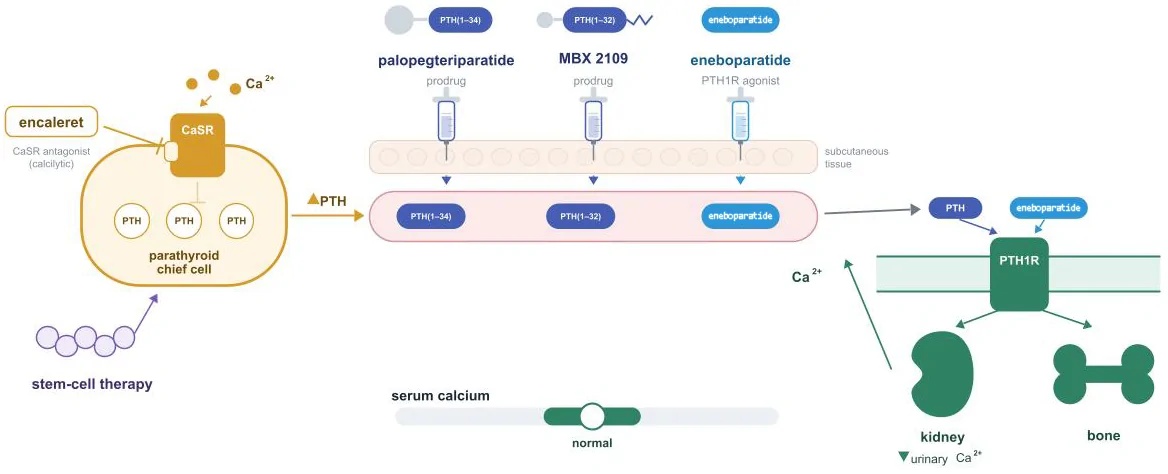
Modes of action of the new therapies.

**Table 1:** Treatment of chronic hypoparathyroidism.

Medication	Dose
Calcium carbonate or calcium citrate	Individualized supplementation. Usually between 1.0 and 3.0 g daily. Preferably with meals to enhance phosphate binding effects.
Calcitriol	The required daily dose is 0.25 to 2.0 µg, administered as a single dose or twice daily.
Alfacalcidol	The required daily dose is 0.5 to 4.0 µg, administered as a single dose.
Cholecalciferol	1000 to 100,000 IU daily based on 25(OH)D level.
Ergocalciferol	50,000 IU weekly to daily based on 25(OH)D level.
Thiazide diuretics (hydrochlorothiazide)	25 to 100 mg daily.

**Table 2:** Monitoring of conventional treatment in patients with chronic hypoparathyroidism.

Parameter	Monitoring Frequency/Recommendations
Serum calcium levels	Twice a year (every 1–2 weeks at the start of treatment or when modifying the dosage of active vitamin D and calcium supplementation).
24-h urinary calcium and creatinine excretion	Every 6 to 24 months.
25(OH)D level	Vitamin D supplementation should be focused on achieving a 25(OH)D level of 75–125 nmol/L (30–50 ng/mL).
Imaging studies of the kidneys, such as ultrasound or computed tomography scans (in patients with history of kidney stones or renal calcifications)	Every 3 to 5 years, or immediately if dysuria occurs.
Blood pressure, ionogram and serum magnesium levels	Regular monitoring in patients receiving thiazide diuretics.
Bone mineral density	It is recommended in postmenopausal women and men above 50 years of age.

**Table 3:** Comparison of conventional therapy and PTH replacement therapy in hypoparathyroidism.

	Conventional Therapy	PTH Replacement Therapy
Calcium and phosphate control	Difficult-to-maintain stable serum calcium levels. Requires regular monitoring of calcium, phosphate, urinary calcium and renal function, as well as frequent dose adjustments. Dietary calcium supplementation and phosphate restriction are often necessary. Increasing doses of active metabolites of vitamin D may raise serum phosphate levels and increase the risk of hyperphosphataemia.	This treatment effectively increases serum calcium levels. More closely restores the physiological actions of PTH and may provide more stable control of calcium and phosphate metabolism. It enabled independence from conventional therapy while maintaining normal serum calcium and phosphate levels.
Hypercalciuria and renal outcomes	High doses of calcium and active vitamin D may increase urinary calcium excretion and contribute to renal complications, including hypercalciuria, nephrolithiasis, nephrocalcinosis and impaired renal function.	This therapy effectively reduces urinary calcium excretion and increases phosphate excretion. It may reduce the need for high doses of calcium and active vitamin D, potentially reducing the burden on the kidneys.
Quality-of-life and pill burden	This treatment does not always provide satisfying quality of life for patients. In certain situations, such as after bariatric surgery, malabsorption disorders or nasogastric tube feeding, conventional therapy may be difficult to implement. Treatment often requires taking large numbers of tablets, which is associated with the risk of side effects and reduced patient comfort. Optimal calcium absorption and stabilisation of calcium and phosphorus levels in blood serum require multiple doses throughout the day.	May reduce the need for oral calcium and active vitamin D and decrease the number of daily tablets. Reduction in treatment burden may contribute to improved patient convenience and quality of life. It is effective, safe and well tolerated in adults with hypoparathyroidism, improving quality of life, physical functioning and well-being. It may reduce the need for oral calcium and active vitamin D, with the greatest reduction in treatment burden observed within the first 3 months, improving convenience and quality of life.
Long-term safety	Long-term treatment requires careful monitoring because of the risk of hypercalciuria, renal complications and ectopic calcifications.	Long-term safety requires continuous monitoring and evaluation of outcomes during treatment.

**Table 4:** Comparison of new and emerging therapies for the treatment of hypoparathyroidism.

Therapeutic Strategy	Mechanism of Action	The Requirement for Exogenous PTH Administration	Endogenous PTH Secretion	Restoration of Parathyroid Tissue	Developmental Status
Palopegteriparatide—recombinant human PTH	PTH prodrug	Yes	No	No	Approved therapy
MBX 2109	PTH prodrug	Yes	No	No	Phase I clinical trial
Eneboparatide—PTH1R receptor agonists	PTH1R activation	No	No	No	Under advanced clinical development (phase III clinical trial)
Encaleret—calcilitic therapy	negative allosteric modulator of CaSR	No	Potentially yes	No	Under advanced clinical development (phase II and III clinical trials)
Autotransplantation	Functional parathyroid tissue	No	Yes	Yes	Clinical/selected use
Allotransplantation	Donor parathyroid tissue	No	Yes	Yes	Experimental/selected use
Stem-cell therapy	Generation of parathyroid-like cells	No	Potentially yes	Potentially yes	Experimental or preclinical approaches (such as other regenerative strategies: microencapsulation, biohybrid implants and gene therapy)

## Data Availability

No new data were created or analyzed in this study. Data sharing is not applicable to this article.
